# Quantification of ^10^B and ^11^B in Biological Samples in Translational Science and Clinical Applications for Boron Neutron Capture Therapy (BNCT)

**DOI:** 10.1002/med.70060

**Published:** 2026-05-15

**Authors:** Christoph Selg, Wolfgang A. G. Sauerwein, Evamarie Hey‐Hawkins

**Affiliations:** ^1^ Centre for Biotechnology and Biomedicine (BBZ), Institute of Bioanalytical Chemistry, Faculty of Chemistry Leipzig University Leipzig Germany; ^2^ Department for Radiation Oncology, Deutsche Gesellschaft für Bor‐Neutroneneinfangtherapie e. V. (DGBNCT) University Hospital Essen Essen Germany; ^3^ Department of Chemistry Babeș‐Bolyai University Cluj‐Napoca Romania

**Keywords:** accelerator‐based neutron sources, boron isotope analysis, clinical dosimetry, mass spectrometry imaging, multimodal molecular imaging, neutron capture spectroscopy, theranostics

## Abstract

Boron neutron capture therapy (BNCT) is a targeted radiotherapy that exploits the selective accumulation of the ^10^B isotope within tumors, followed by irradiation with low‐energy neutrons to induce high linear energy transfer particles with short path lengths, resulting in localized tumor cell destruction while sparing surrounding healthy tissue. Achieving optimal therapeutic efficacy requires precise quantification of ^10^B and ^11^B in biological samples such as blood, tissue, and cells to inform treatment planning, dosimetry, and patient selection. This review provides a comprehensive assessment of analytical methods for boron (isotope) determination in BNCT, covering neutron‐based techniques, mass spectrometry, nuclear imaging, and spectroscopic and magnetic approaches. Each method is discussed in terms of analytical principles, sample preparation, sensitivity, isotopic specificity, invasiveness, real‐time capability, infrastructure complexity, and clinical applicability. Particular emphasis is placed on spatially resolved and in vivo techniques, as well as emerging theranostic strategies that integrate boron delivery with multimodal imaging. Despite significant technological progress, the lack of widely available, real‐time, non‐invasive, and ^10^B‐specific dosimetry remains a key limitation for routine clinical implementation. In particular, while positron emission tomography‐based approaches such as 4‐borono‐2‐[^18^F]fluoro‐L‐phenylalanine provide essential information on boron biodistribution for treatment planning, they reflect tracer‐level pharmacokinetics and therefore offer indirect rather than absolute quantification of therapeutic boron concentrations. This review critically assesses current methodologies in the context of clinical readiness and outlines future directions to support the translation of BNCT from bench to bedside.

**Abbreviations**
^18^F‐FBPA: 4‐borono‐2‐[^18^F]fluoro‐L‐phenylalanine
AG: annihilation γ‐rays
BNCT: boron neutron capture therapy
BODIPY: boron‐dipyrromethene
BPA: 4‐borono‐L‐phenylalanine
BPA‐F: BPA‐fructose complex
BSA: bovine serum albumin
BSH: sodium mercaptoundecahydro‐*closo*‐dodecaborate
CBE: compound biological effectiveness
CD98: 4F2 heavy chain (SLC3A2)
CPAA: charged particle activation analysis
CR‐39: poly(allyl diglycol carbonate) (PADC)
CZT: cadmium zinc telluride
DCP‐AES: direct current plasma atomic emission spectrometry
DiAmSar: 1,8‐diamino‐3,6,10,13,16,19‐hexaazabicyclo[6.6.6]icosane (sarcophagine)
DNA: deoxyribonucleic acid
DOTA: 1,4,7,10‐tetraazacyclododecane‐1,4,7,10‐tetraacetic acid
DOTA‐JR11: DOTA‐conjugated somatostatin receptor antagonist JR11
DOTA‐TATE: DOTA‐(Tyr3)‐octreotate
EELS: electron energy loss spectroscopy
ESR/EPR: electron spin resonance/electron paramagnetic resonance
EXAFS: extended X‐ray absorption fine structure
FIB‐MS: focused ion beam mass spectrometry
FNTD: fluorescent nuclear track detector
FT‐IR: Fourier‐transform infrared spectroscopy
GAGG: gadolinium aluminum gallium garnet scintillator
GBM: glioblastoma multiforme
HPGe: high‐purity germanium
HRQAR: high‐resolution quantitative alpha autoradiography
ICP‐AES/OES: inductively coupled plasma atomic/optical emission spectrometry
ICP‐MS: inductively coupled plasma mass spectrometry
IG‐IMRT: image‐guided intensity‐modulated radiation therapy
IMRT: intensity‐modulated radiation therapy
J‐PET: Jagiellonian PET
i‐TED: total energy detector Compton camera
LaBr_3_(Ce+Sr): lanthanum bromide scintillator doped with cerium and strontium
LA‐ICP‐MS: laser ablation inductively coupled plasma mass spectrometry
LAT1: L‐type amino acid transporter 1
LIBS: laser‐induced breakdown spectroscopy
LYSO: lutetium‐yttrium oxyorthosilicate
MALDI‐MSI: matrix‐assisted laser desorption/ionization mass spectrometry imaging
MRI: magnetic resonance imaging
MS: mass spectrometry
MSI: mass spectrometry imaging
NCR: neutron capture radiography
NIRF: near‐infrared fluorescence
NIS: sodium‐iodide symporter
NMR: nuclear magnetic resonance
NP: nanoparticle
OES: optical emission spectrometry
PADC: poly(allyl diglycol carbonate)
PET: positron emission tomography
PG: prompt γ‐rays
PGAA/PGNAA: prompt γ‐ray (neutron) activation analysis
PGRA/PGRS: prompt γ‐ray analysis/spectroscopy
PG‐SPECT: prompt γ single‐photon emission computed tomography
PIGE: particle‐induced γ‐ray emission
PIXE: particle‐induced X‐ray emission
PLGA: poly(lactic‐co‐glycolic acid)
QNCR: quantitative neutron capture radiography
RGD: arginine–glycine–aspartic acid
SIMS: secondary ion mass spectrometry
SiPM: silicon photomultiplier
SNMS: secondary neutral mass spectrometry (laser post‐ionization)
SPECT: single‐photon emission computed tomography
STIM: scanning transmission ion microscopy
STXM: scanning transmission X‐ray microscopy
SWIR: short‐wave infrared
T/B: tumor‐to‐blood
TEM: transmission electron microscopy
T/N: tumor‐to‐normal tissue ratio
ToF‐SIMS: time‐of‐flight secondary ion mass spectrometry
UHPLC‐MS/MS: ultra‐high‐performance liquid chromatography coupled with tandem mass spectrometry
UV: ultraviolet
VMAT: volumetric modulated arc therapy
XANES: X‐ray absorption near‐edge structure
XAS: X‐ray absorption spectroscopy

## 1 Introduction

The global incidence of cancer continues to increase, particularly for cases involving recurrent, inoperable, or treatment‐resistant tumors. Cancer remains the second leading cause of death worldwide, with approximately 9.7 million fatalities each year in 2022, corresponding to nearly 10 million deaths globally, with a continuing upward trend [1, 2]. While chemotherapy and surgery remain the mainstays of cancer treatment, many patients experience recurrence or show poor response to conventional therapies. This has driven the development of more selective and less toxic alternatives aimed at improving survival outcomes [3, 4]. In this context, boron neutron capture therapy (BNCT) has emerged as a promising modality, particularly for radioresistant and recurrent tumors such as head and neck cancers, glioblastoma, and melanoma, where recent accelerator‐based clinical studies have demonstrated encouraging results [5].

**Figure 1:**
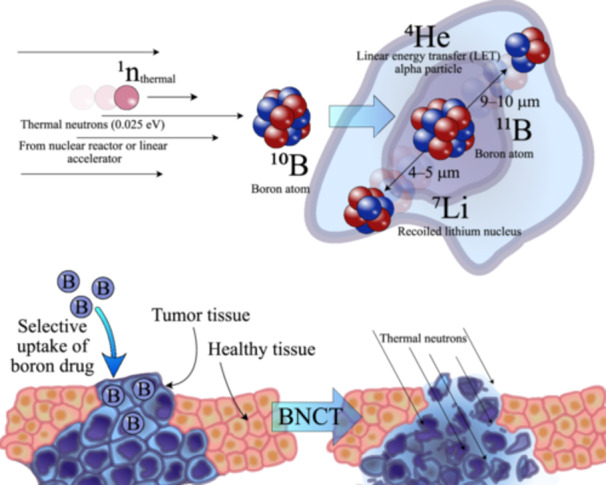
Top: Physical principle of BNCT and mechanism of action at the cellular level. Bottom: Schematic illustration of BNCT selectivity at the tissue level, showing preferential accumulation of ^10^B in tumor cells (blue) over healthy cells (apricot). [Color figure can be viewed at wileyonlinelibrary.com]

BNCT is a binary therapeutic strategy consisting of two key components (Figure 1): First, ^10^B is selectively delivered to tumor tissues using specialized delivery agents. Second, the tumor area is irradiated with low‐energy neutrons [6, 7].

When a neutron is captured by a ^10^B nucleus, it forms an unstable ^11^B* nucleus that rapidly undergoes fission: ^10^B + ^1^n → ^7^Li + ^4^He. This reaction releases a densely ionizing α‐particle (^4^He, ~150 keV/µm) and a ^7^Li nucleus (~175 keV/µm), along with 2.31 MeV of kinetic energy and weak γ‐radiation [8, 9, 10]. The high linear energy transfer (LET; the amount of energy deposited along a radiation track) of these particles and their limited range of about 5–9 µm [5], corresponds approximately to the diameter of a single cell. This results in complex or clustered DNA damage, defined as multiple lesions occurring within one or two turns of the DNA helix [11, 12]. This type of damage is more difficult for cells to repair, resulting in a higher relative biological effectiveness for BNCT compared to low‐LET radiation like X‐rays or γ‐rays, thereby enhancing tumor cell kill [9, 13].

The selectivity of BNCT arises from this localized cytotoxic effect, combined with the preferential accumulation of ^10^B in tumor cells, and enables precise targeting of malignant tissue at the cellular level while minimizing damage to adjacent healthy tissue [5]. Atoms vary in their likelihood of capturing thermal neutrons, a property described by their neutron capture cross‐section (measured in barns, where 1 barn is 10^−28^ m^2^, stating the cross‐sectional area of nuclei and nuclear reactions) [14]. Elements that make up human tissue, such as hydrogen, carbon, nitrogen, and oxygen, have low cross‐sections (0.33, 0.0035, 1.9, and 0.00019 barns) and are therefore unlikely to undergo neutron capture [15]. ^10^B, with a cross‐section of 3840 barns, is the most viable candidate due to its favorable biological properties [16]. Natural boron consists of two stable isotopes: approximately 20% is ^10^B, the therapeutically active species in BNCT, while the remaining 80% is ^11^B. The ^11^B isotope exhibits a neutron capture cross‐section of only about 0.005 barns, making it effectively inert under therapeutic neutron fluxes. For this reason, boron delivery agents used in BNCT must be enriched in ^10^B. Furthermore, an effective delivery agent must fulfill several essential criteria. It should selectively accumulate within cancer cells, ideally delivering at least 10^9 10^B atoms per cell or achieving a concentration of 25–30 µg per gram of tumor tissue. Minimal uptake in healthy tissue and blood is critical to avoid systemic toxicity and to preserve the therapeutic selectivity of BNCT. The tumor/normal tissue (T/N) and tumor/blood (T/B) ratio of ^10^B should be at least 4:1, with higher ratios preferred [10]. Additional properties include non‐toxicity in the absence of neutron exposure and sufficient solubility in aqueous media to allow for intravenous administration [17, 18]. The design and evaluation of boron delivery agents is an extensive research area and is not the focus of this review. Readers are referred to dedicated publications for comprehensive discussions on this topic [16, 19, 20, 21, 22, 23, 24, 25].

The effectiveness of BNCT relies on multiple factors, including the selective uptake and retention of ^10^B in malignant tissue, the timing of neutron irradiation, and the precise energy profile of the neutron beam. Early clinical studies occasionally failed to demonstrate clear therapeutic benefits, in part because thermal neutrons lost energy during tissue penetration and fell below the threshold necessary for nuclear capture before reaching the tumor.

To address this issue, surgical exposure of tumors to the neutron beam was initially explored, though it significantly increased procedural complexity [26]. A more practical and effective solution was the introduction of epithermal neutrons, which lose energy on the way through tissue and reach thermal levels at the site of the tumor [27].

Historically, BNCT relied on nuclear reactors as the primary neutron source, and considerable efforts have been made to adapt these systems for therapeutic applications. While technically functional, these reactors suffered from limitations in neutron energy consistency and accessibility. Many were situated in remote research facilities, far removed from clinical settings, which hindered patient access and slowed clinical development. These challenges discouraged widespread adoption and contributed to inconclusive trial results regarding the efficacy of boron delivery agents [28, 29]. Recent work has demonstrated that existing research reactors can be successfully modified for BNCT applications; for example, adaptation of the radial beam port of the ITU TRIGA Mark‐II reactor enabled the production of epithermal neutrons and their characterization for dosimetric and biological studies [30].

The field has since undergone a transformative shift. The development of accelerator‐based neutron sources has eliminated many of the logistical barriers associated with reactor‐based systems [7]. These compact, hospital‐compatible devices provide more consistent neutron fluxes and are now operational in clinical settings [31]. Japan and other countries in Asia have installed such systems and have already reported clinical results [32]. These advances have reignited interest in BNCT and are enabling broader integration into oncology practice by overcoming one of its most significant logistical bottlenecks [31, 33, 34]. While these technological advances are essential to the therapy's transition from experimental to routine clinical use, a detailed discussion of neutron source development falls outside the scope of this review and is addressed comprehensively elsewhere [7, 23, 33, 35, 36, 37, 38].

As BNCT enters the clinical arena, attention must now turn to the critical role of boron quantification. Determining the spatial and temporal distribution of ^10^B (and ^11^B) in biological matrices is essential for treatment planning, dosimetry, and therapy monitoring. This requires sensitive, reliable, and clinically feasible analytical techniques that are compatible with real‐world diagnostic workflows. While numerous approaches have been developed, their clinical translatability remains variable [23, 39, 40, 41, 42].

This review provides a comprehensive assessment of analytical and imaging techniques for boron quantification in biological samples. Particular focus is placed on sample preparation, analytical sensitivity, and clinical applicability. By evaluating these methods within the framework of translational science, this review aims to support the continued evolution of BNCT from a specialized experimental therapy to a fully integrated component of modern cancer treatment.

## 2 Literature Search Strategy

This review is based on a structured, narrative analysis of the literature on boron quantification methods in BNCT. An initial set of key publications, including foundational reviews and monographs in the field, was used to establish the conceptual framework. From these sources, relevant literature was identified through backward citation tracking and expanded using databases such as SciFinder, CrossRef, and Google Scholar.

The literature was iteratively organized into methodological categories using Citavi 6 corresponding to analytical techniques applicable to biological samples in BNCT. Additional targeted searches were performed within each category to identify the most recent developments, with a particular focus on studies addressing boron quantification in biological matrices and their relevance for translational and clinical applications.

Both preclinical and clinical studies were considered, with prioritization on work demonstrating analytical relevance, methodological innovation, or clinical applicability. Established but outdated or less clinically relevant techniques were included for completeness, but discussed in less detail. The final structure of the review reflects this iterative categorization and evaluation process.

## 3 Analytical Requirements in BNCT

**Figure 2:**
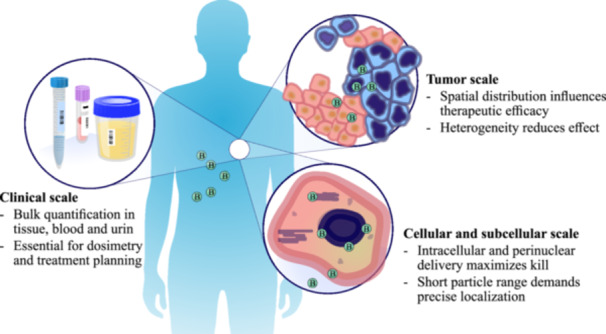
Illustration of the spatial scales relevant to boron quantification in BNCT. Clinical‐scale measurements (whole‐body, blood or urine sampling), tumor‐scale imaging (regional and intratumoral distribution), and cellular‐ or subcellular‐scale analysis (intracellular and perinuclear localization) are shown. Green circles marked with “B” represent boron‐containing drugs, highlighting how concentration and localization vary across scales. While blood‐based dosimetry remains the clinical standard in many BNCT protocols, it may underestimate intratumoral heterogeneity, as demonstrated by recent ^18^F‐FBPA PET and PG‐SPECT studies. [Color figure can be viewed at wileyonlinelibrary.com]

The therapeutic effect of BNCT depends critically on the presence, concentration, and localization of ^10^B within tumor cells. Because of the limited range of the particles generated by the neutron capture reaction, maximum efficacy is only achieved when ^10^B is delivered intracellularly and preferably near the cell nucleus (perinuclear). This makes the accurate quantification of boron in tissues, blood, and other biological fluids a fundamental requirement for treatment planning and dosimetry. Equally important is the ability to assess the microscopic and subcellular distribution of boron within the tumor microenvironment. Traditional reliance on blood measurements to estimate tumor boron content using fixed ratios introduces considerable uncertainty and may not adequately capture inter‐ and intratumoral heterogeneity (Figure 2) [23, 40, 41, 42]. Recent studies using nuclear imaging techniques such as 4‐borono‐2‐^18^F‐fluoro‐L‐phenylalanine (^18^F‐FBPA) PET further highlight this limitation by demonstrating heterogeneous boron distribution within tumors that is not reflected by blood‐based measurements (see Section 5.5).

The pharmacokinetics and spatial distribution of boron compounds vary depending on tumor cellularity, cell cycle stage, and the physicochemical properties of the delivery agent. Incomplete or uneven uptake across the tumor mass can compromise therapeutic outcomes [43]. Therefore, accurate measurement methods should support both bulk quantification and three‐dimensional imaging of boron distribution within the body. Ideally, these methods should be minimally invasive, capable of real‐time or near‐real‐time operation, and adaptable to clinical timelines for decision‐making. In the context of BNCT, the terms “real‐time” and “near‐real‐time” are used to describe the temporal relationship between measurement and clinical decision‐making. “Real‐time” refers to analytical methods that provide continuous or quasi‐continuous feedback during neutron irradiation or within the treatment session itself, thereby enabling immediate assessment of boron distribution and potentially adaptive intervention. In contrast, “near‐real‐time” denotes methods that deliver results within a clinically actionable timeframe, typically on the order of minutes to a few hours, allowing integration into treatment planning decision workflows, but not direct in situ monitoring during irradiation. Methods requiring longer processing times (e.g., extensive sample preparation or off‐site analysis) are considered offline and are primarily used for retrospective validation rather than immediate clinical guidance. These definitions are applied consistently throughout this review to enable a more standardized comparison of analytical techniques. Imaging at the cellular and subcellular levels is particularly valuable for assessing drug behavior, validating dosimetry models, and supporting the development of new delivery agents [23, 44, 45, 46].

Current clinical BNCT protocols require repeated blood sampling before, during, and after neutron irradiation to dynamically monitor boron concentration, as this directly influences dosimetry and patient‐specific treatment planning. However, conventional techniques are frequently insufficient for timely, in situ quantification, and the absence of reliable real‐time methods remains a significant limitation. Advancing BNCT requires the integration of analytical tools that not only measure boron concentrations with high accuracy and spatial resolution but also enable patient‐specific dosimetric planning. As such, the development and clinical validation of advanced analytical and imaging technologies are essential for the continued success and expansion of BNCT.

## 4 Sample Preparation Methodologies

**Figure 3:**
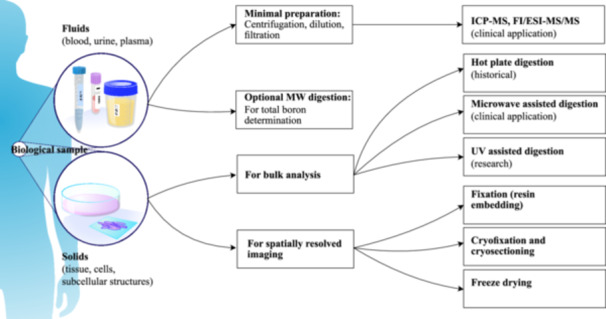
Sample preparation strategies for boron quantification in BNCT. Fluid samples (blood, plasma, urine) generally require minimal processing and are directly compatible with clinical mass spectrometry workflows. Solid samples (tissue, cells, subcellular structures) need more elaborate preparation, such as fixation, cryosectioning, for spatially resolved imaging or digestion for bulk analysis. [Color figure can be viewed at wileyonlinelibrary.com]

Accurate quantification of ^10^B and ^11^B in biological matrices requires meticulous sample preparation tailored to the analytical technique and biological matrix. Proper handling must preserve analyte distribution and integrity while enabling reproducibility and compatibility with sensitive instrumentation given boron's memory effects and mass discrimination in complex biological matrices. The nature of the biological matrix of the sample types in BNCT studies range from blood and plasma to solid tumor tissue and subcellular structures and dictates the complexity of the preparation required (Figure 3).

Fluid samples, such as blood and urine, typically undergo minimal preparation, involving centrifugation or dilution, and are directly compatible with mass spectrometry (MS)‐based techniques, such as flow injection/electrospray ionization tandem mass spectrometry (FI/ESI‐MS/MS) and inductively coupled plasma mass spectrometry (ICP‐MS) [47, 48]. In contrast, tissue and cellular samples require more complex processing to ensure analyte stability and spatial integrity [49, 50]. For imaging and spatially resolved analyses, fixation and embedding are commonly employed to maintain tissue structure. Chemical fixation using glutaraldehyde or formaldehyde preserves structural morphology for imaging but can introduce boron contamination or redistribution. Embedding tissues in epoxy or acrylic resins supports sectioning for secondary ion mass spectrometry (SIMS), electron energy loss spectroscopy (EELS), and autoradiography. However, some resins may interfere with elemental analysis, requiring thorough validation [40, 51]. When preservation of native boron distribution is essential, cryofixation and cryosectioning offer superior spatial fidelity. Cryosectioning and cryofixation are critical for spatially resolved techniques like nanoSIMS and laser postionization secondary neutral mass spectrometry (laser‐SNMS). Rapid freezing minimizes diffusion and preserves in situ boron localization. This approach provides high spatial fidelity, but it requires cryostats and high‐vacuum compatibility, making it more suitable for research settings [49, 51].

To stabilize biological samples and enable long‐term storage, freeze‐drying (lyophilization) is often employed.

Freeze‐drying stabilizes samples by sublimating water under vacuum. It is commonly used prior to nanoSIMS, EELS, and laser‐based ion beam techniques. It helps prevent boron leaching and supports long‐term storage, but demands careful control to avoid structural distortion [48, 50, 51] For bulk elemental analysis, various digestion methods are widely used (Table 1). The main approaches include:

– *Hot‐plate digestion* with nitric or mixed acids was employed in early BNCT studies for quantitative analysis using ICP atomic emission spectrometry (ICP‐AES) and direct current plasma AES (DCP‐AES). However, this approach can lead to boron loss through volatilization as boric acid esters and often yields variable recovery. In addition, complete decomposition of complex boron‐containing compounds is not always achieved, as polyhedral boranes and carboranes can resist breakdown under hot nitric acid conditions. When boron agents are conjugated to carriers or encapsulated in liposomes, their structural stability may further hinder digestion and compromise quantitative accuracy [40, 52].
– *Microwave‐assisted* digestion, which offers controlled pressure and temperature for efficient tissue breakdown, is widely regarded for its speed and reproducibility [48, 50, 53, 54].
– *Ultraviolet (UV)‐assisted digestion* employs ultraviolet radiation in combination with oxidizing agents such as H_2_O_2_ to decompose organic matter, making it suitable for sensitive applications, although generally limited to low sample throughput. In combination with hot‐plate‐assisted acid digestion, however, the method has proven highly effective for preparing biological samples for boron determination at low μg L^−1^ concentrations by ICP‐MS. This combined protocol efficiently removes carbon, minimizing signal suppression or enhancement, and maintains low background boron levels, resulting in a detection limit as low as 0.4 μg L^−1^ with complete recovery of boron from cell samples [54, 55].

**Table 1:** Comparison of digestive sample preparation methods used for bulk elemental analysis in BNCT.

Method	Principles and reagents	Advantages	Limitations	Typical BNCT application	Clinical readiness	References
Hot‐plate acid digestion	Open‐vessel heating with nitric or mixed acids; oxidizes organics and dissolves tissues for ICP‐AES/DCP‐AES	Simple equipment; historically common in BNCT studies	Risk of B loss via volatilization (boric acid esters); variable recovery; slower digestion times	Early boron quantification in blood/tissue	Low (largely superseded; variable recovery)	[40, 52]
Microwave‐assisted digestion	Closed‐vessel digestion with nitric or mixed acids under controlled pressure/temperature	Fast; reproducible; efficient tissue breakdown; high throughput; minimizes contamination	Requires microwave digestion unit; higher initial cost	Modern clinical workflows for blood/tissue B analysis	High (established in clinical/toxicology labs)	[48, 53]
UV‐assisted digestion	UV radiation and oxidizing agent (e.g., H_2_O_2_) to photodecompose organics	Minimal contamination risk; gentle on analytes; suitable for trace/sensitive work	Low throughput; slow; limited sample size	Specialized research with ultra‐low boron levels	Low–moderate (specialized research applications)	[54, 55]

Each method must be optimized to minimize boron contamination and volatilization. Closed‐vessel microwave systems, in particular, have shown excellent recovery rates and are already employed in clinical environments for trace element analysis and toxicology [52].

For imaging techniques such as quantitative neutron capture radiography (QNCR), tissues are embedded in polymethyl methacrylate or acrylic resins. These enable three‐dimensional visualization of boron distributions but may introduce scattering artifacts and are currently limited to research applications [49, 51].

In clinical settings, preparation methods must meet stringent criteria for safety, speed, and reproducibility. Blood and plasma analysis after minimal processing is the most established approach. Microwave‐assisted digestion offers a balance between precision and operational feasibility, making it suitable for clinical workflows. Conversely, cryogenic methods, freeze‐drying, and embedding techniques remain primarily confined to translational or preclinical research due to their complexity, equipment needs, and time requirements. A general challenge across all digestion strategies is the chemical robustness of boron clusters such as polyhedral boranes and carboranes, which can resist complete breakdown and thereby compromise quantitative recovery if not carefully optimized [49, 50, 53].

## 5 Analytical Techniques for Boron Quantification in BNCT

A broad range of analytical techniques for the measurement of ^10^B and ^11^B in biological systems has been developed to quantify boron concentrations, assess biodistribution, and support dosimetry. These techniques differ significantly in sensitivity, spatial resolution, invasiveness, infrastructure requirements, and clinical applicability. In this section, we introduce and categorize the techniques into functional groups based on their physical principles. Each method is then evaluated individually, covering analytical principles and instrumentation, sample preparation requirements, sensitivity, specificity, resolution, differentiation between ^10^B and ^11^B, invasiveness, real‐time capability, infrastructure complexity, destructiveness, clinical suitability, and integration into clinical workflows. In general, effective microanalysis of living samples requires several conditions: minimal sample volume, avoidance of pre‐treatment steps, minimal interference from other elements, and short measurement durations sufficient to achieve the desired accuracy [49, 51, 56].

### 5.1 Overview and Categorization of Techniques

**Figure 4:**
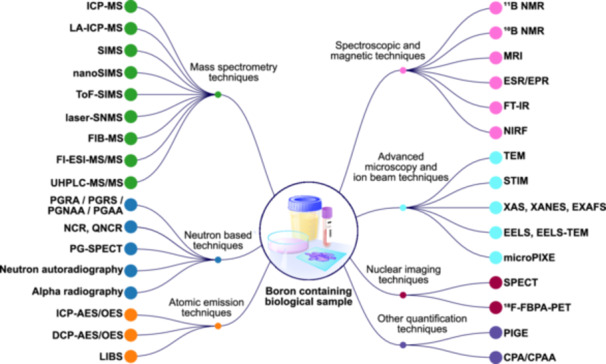
Overview of analytical methods applied in BNCT research, organized by major technique families. The dendrogram reflects the classification structure used in Chapter 4, grouping neutron‐based, atomic emission spectrometric, mass spectrometry‐based, nuclear imaging, spectroscopic, magnetic, advanced microscopy, ion beam, and other quantitative techniques. Only representative and widely used abbreviations are shown; full definitions are provided in the text. [Color figure can be viewed at wileyonlinelibrary.com]

Given the wide range of analytical techniques applied in BNCT, a systematic classification is useful to highlight their underlying principles and areas of application. The methods discussed in this section are therefore grouped into major categories, as illustrated in Figure 4.

#### 5.1.1 Neutron‐Based Techniques

Prompt gamma‐ray analysis or spectroscopy (PGRA or PGRS), also referred to as prompt gamma neutron activation analysis (PGNAA) or prompt gamma‐ray activation analysis (PGAA), prompt gamma single‐photon emission computed tomography (PG‐SPECT or BNCT‐SPECT), neutron capture radiography (NCR), QNCR, alpha autoradiography, and high‐resolution quantitative alpha autoradiography (HRQAR).

#### 5.1.2 AES Techniques

ICP atomic/optical emission spectrometry (ICP‐AES/OES), DCP‐AES, and laser‐induced breakdown spectroscopy (LIBS).

#### 5.1.3 MS‐Based Techniques

ICP‐MS, mass spectrometry imaging (MSI), SIMS, laser‐SNMS, nanoSIMS, time‐of‐flight SIMS (ToF‐SIMS), laser ablation ICP‐MS (LA‐ICP‐MS), focused ion beam mass spectrometry (FIB‐MS), matrix‐assisted laser desorption/ionization mass spectrometry imaging (MALDI‐MSI), and ultra‐high‐performance liquid chromatography coupled with tandem mass spectrometry (UHPLC‐MS/MS).

#### 5.1.4 Nuclear Imaging Techniques

SPECT and positron emission tomography (PET; especially ^18^F‐FBPA‐PET).

#### 5.1.5 Spectroscopic and Magnetic Techniques

Nuclear magnetic resonance (^10^B NMR, ^11^B NMR), magnetic resonance imaging (MRI), fluorescence spectroscopy, electron spin resonance/electron paramagnetic resonance (ESR/EPR), Fourier‐transform infrared spectroscopy (FT‐IR), and near‐infrared fluorescence (NIRF).

#### 5.1.6 Advanced Microscopy and Ion Beam Techniques

Transmission electron microscopy (TEM), scanning transmission ion microscopy (STIM), X‐ray absorption spectroscopy (XAS), EELS, and particle‐induced X‐ray emission with micrometer‐sized beams (micro‐PIXE).

#### 5.1.7 Other Quantitative Techniques

Particle‐induced gamma‐ray emission (PIGE) and charged particle activation analysis (CPA or CPAA).

### 5.2 Neutron‐Based Techniques

Neutron‐based analytical methods are among the most specific techniques available for detecting boron in biological matrices. These methods harness the nuclear reactions resulting from neutron capture to produce prompt γ‐radiation. While these techniques offer unparalleled specificity and, in some cases, spatial resolution, their dependency on neutron sources (traditionally nuclear research reactors) has historically restricted their use to specialized research environments. However, with the advent of hospital‐based accelerator‐driven neutron sources, their future clinical applicability may expand significantly [23, 57].

**Figure 5:**
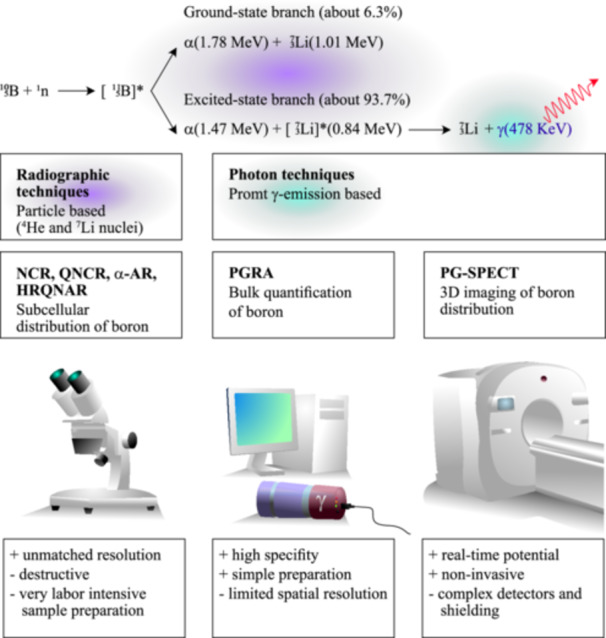
Neutron capture by ^10^B produces α‐particles (^4^He^2+^), ^7^Li nuclei, and a characteristic 478 keV γ‐quantum. This γ‐emission forms the basis for neutron‐based analytical methods in BNCT, including PGRA for bulk quantification, PG‐SPECT for three‐dimensional imaging, and autoradiography for subcellular resolution, each with distinct advantages and limitations. [Color figure can be viewed at wileyonlinelibrary.com]

PGRA or PGRS, also referred to as PGNAA or PGAA, is one of the most established techniques for quantifying ^10^B concentrations in BNCT research. This method detects the characteristic 478 keV gamma photon emitted from the excited ^7^Li* nucleus, produced in the ^10^B(*n*,α)^7^Li* capture reactions (Figure 5). Early applications of PGRA were primarily limited to bulk measurements of biological tissues using high‐purity germanium (HPGe) detectors within thermal neutron beams. The detection thresholds in these settings ranged from 0.1 to 0.5 ppm of ^10^B, and sample preparation was minimal, typically involving simple enclosure in boron‐free Teflon containers [56, 58, 59, 60, 61, 62, 63].

Recent developments have significantly enhanced PGRA's scope, including the introduction of LaBr_3_(Ce+Sr)‐based scintillation detector systems read out by silicon photomultipliers (SiPMs). This configuration successfully resolved the 478 keV peak even under background gamma radiation and demonstrated linear quantification of ^10^B concentrations down to 62 ppm under a neutron flux of 10^5^ cm^−2^ s^−1^ [64]. Furthermore, Okazaki et al. developed an 8 × 8 LaBr_3_(Ce) scintillator array coupled to multi‐pixel photon counters, demonstrating limited spatial resolution and stable prompt gamma‐detection performance suitable for BNCT beamlines [65]. Caracciolo et al. further advanced their LaBr_3_(Ce+Sr)‐SiPM modules, achieving robust 478 keV peak resolution under clinically relevant background conditions and enabling spatial localization of the boron signal [66]. Nutter et al. explored the use of a modified LaBr_3_(Ce) scintillator array as a Compton camera, showing the potential for real‐time imaging of boron distributions during neutron irradiation [67]. Complementing these efforts, Silarski et al. proposed a position‐sensitive scintillator system with anti‐Compton shielding, aimed at in vivo PGRA monitoring with improved background suppression [68]. Collectively, these developments point toward more compact, sensitive, and clinically adaptable PGRA instrumentation for BNCT dosimetry and treatment verification. PGRA, in combination with ICP‐AES and complementary imaging methods, has been successfully applied to quantify ^10^B concentrations in bone tissue following 4‐borono‐L‐phenylalanine (BPA) administration, enabling the determination of compound biological effectiveness (CBE) factors for BNCT‐related bone damage assessment [69]. The feasibility of hospital‐based PGRA has been advanced both by the development of in‐hospital systems, such as the prototype installed at the In‐Hospital Neutron Irradiator in Beijing, and by the introduction of accelerator‐based neutron sources that enabled BNCT implementation in clinical facilities in Japan [51, 70]. Complementary work on detector modeling and optimization has further refined PGRA's quantitative accuracy and supports its translation into routine clinical workflows [71].

Nonetheless, the technique still faces challenges: PGRA requires access to a dedicated neutron source, shielding infrastructure, and often prolonged acquisition times when targeting low boron concentrations. These constraints limit its routine clinical deployment, though its strengths in isotope specificity, insensitivity to effects of the sample matrix, no complex sample preparation (e.g., digesting the sample), and quantitative accuracy ensure continued relevance in both preclinical validation and translational research.

In an effort to bridge the gap between quantitative accuracy and spatial resolution, PGRA has been extended into its tomographic form, known as PG‐SPECT or BNCT‐SPECT. While conventional PGRA enables bulk quantification of ^10^B through detection of the characteristic 478 keV photons emitted by the decay of excited ^7^Li* nuclei after the ^10^B(*n*,α)^7^Li* reaction, its scope is largely limited to point or planar measurements. PG‐SPECT advances this principle by acquiring tomographic data from the same prompt gamma emissions, thereby enabling three‐dimensional reconstruction of ^10^B distribution in vivo. While this section (Section 5.2) focuses on the technical development and feasibility of PG‐SPECT as a neutron‐based method, its positioning among conventional nuclear imaging modalities is addressed in Section 5.5, and its potential role within future theranostic and multimodal strategies is further discussed in Section 6.

Feasibility studies and prototype experiments have demonstrated the conceptual potential of PG‐SPECT, laying the groundwork for further technical development, but not yet for validated clinical deployment. In the BNCT treatment room, many X‐ and γ‐rays are emitted in addition to the prompt γ‐rays (PG) emitted by boron neutron capture reactions. Among them, annihilation γ‐rays (AG) have an energy of 511 keV, and the energy difference with PGs of 478 keV is only 33 keV (approximately 7%). To distinguish the PG from the AG with an energy‐sensitive detector, high‐energy resolution detection is necessary. Consequently, these early systems faced challenges in resolution, sensitivity, and background suppression due to the high‐energy photon environment and overlap with the 511 keV annihilation peak [72, 73, 74]. A major translational challenge remains the high gamma background in BNCT treatment environments, including contributions from shielding materials and facility design, which complicates reliable prompt gamma detection under real treatment conditions.

More recent advances have significantly improved the technical performance of PG‐SPECT prototypes, including a system utilizing cadmium zinc telluride (CdZnTe or CZT) detectors in combination with bismuth germanate anti‐Compton suppression modules. This configuration successfully reconstructed 478 keV γ‐ray distributions from phantom models with high fidelity and extrapolated their performance under clinical fluences, with overestimation and deviation remaining within acceptable margins for dosimetric application [75].

Subsequently, Kanno et al. proposed a novel current‐mode PG‐SPECT approach based on a transXend detector system. By measuring prompt γ‐emissions as induced electric currents rather than through conventional photon counting and applying neural network analysis to account for attenuation effects, their simulation study demonstrated the feasibility of reconstructing boron distributions with clinically relevant concentration ranges [76].

The feasibility of real‐time boron monitoring was explored in pilot experiments at the ILL‐Grenoble reactor using the high‐efficiency i‐TED Compton camera. In this setup, cell samples from tongue squamous cell carcinoma, malignant melanoma, and glioblastoma treated with BPA were irradiated with a thermal neutron spectrum, and the resulting 478 keV photons from ^7^Li de‐excitation were recorded both with the Compton imager and a high‐sensitivity HPGe array. These experiments demonstrated the imaging capability of the Compton device for the 478 keV line and confirmed sufficient sensitivity for monitoring boron uptake, thereby underscoring its potential for online dose verification during treatment [77]. Murata et al. advanced the development of BNCT‐SPECT systems through innovative detector designs (CdTe and later GAGG), optimized collimator configurations, and energy‐resolution improvements that enabled discrimination of 478 keV capture γ‐rays from background radiation. Their design studies and prototypes achieved approximately 5 mm spatial resolution and 5% quantitative accuracy under controlled conditions, supporting the technical feasibility of PG‐SPECT for treatment‐integrated BNCT dosimetry, while further validation in realistic clinical environments remains necessary [57, 78, 79, 80, 81].

PG‐SPECT systems tailored specifically for BNCT have been highlighted in reviews as promising tools to provide real‐time information on the macroscopic average boron dose delivered to patients, representing an important step toward treatment‐integrated dosimetry [81].

Collectively, these innovations establish PG‐SPECT as a technically promising approach for treatment‐integrated boron monitoring in BNCT. Its potential advantages include three‐dimensional, non‐invasive assessment of boron distribution during irradiation. However, the current evidence base is still largely limited to prototypes, phantom studies, simulations, and pilot experiments, and substantial challenges remain with regard to background suppression, facility integration, and validation under routine clinical conditions. Accordingly, PG‐SPECT should presently be regarded as an emerging translational technology rather than a clinically established method.

Complementary to this macroscopic imaging technique, autoradiographic methods provide critical insight into the subcellular distribution of boron in biological samples. Neutron autoradiography leverages the spatial detection of charged particles emitted during the ^10^B(*n*,α)^7^Li reaction. Following neutron irradiation, the boron‐containing samples are placed in contact with charged‐particle‐sensitive detectors, typically CR‐39 (poly(allyl diglycol carbonate) or PADC) plastic films. These films are chemically etched post‐irradiation to visualize the particle tracks, which can then be quantified through image analysis and correlated with ^10^B concentration via pre‐established calibration curves [40, 82]. Recent work has refined neutron autoradiography by combining it with UV‐C imprinting on PADC detectors, enabling visualization of cellular outlines alongside particle tracks. Applied to BPA‐treated glioblastoma cells, the method allowed discrimination of boron distribution between nucleus and cytoplasm, providing improved subcellular resolution for boron drug evaluation [83]. While early neutron autoradiography was primarily qualitative, later methodological refinements enabled QNCR. By introducing calibrated track detectors and optimized etching protocols, the technique was adapted to provide reliable boron concentration measurements in both liquid and tissue samples. Cross‐validation with complementary analytical methods confirmed its accuracy, and subsequent applications in tumor models demonstrated its suitability for evaluating both established boron carriers, such as BPA, and experimental formulations. These developments marked the transition of autoradiography from a purely descriptive imaging approach to a semi‐quantitative and, in some cases, fully quantitative tool for biodistribution studies [82, 84]. By providing spatially resolved information on boron distribution within heterogeneous tissue samples, autoradiographic methods overcome the limitations of bulk uptake measurements, which only yield averaged values. This capability is particularly relevant for distinguishing tumor from normal tissue in mixed specimens, thereby supporting the validation of boron carriers and improving confidence in dosimetric assessments [85].

A complementary autoradiographic approach combines the CR‐39 track detection with optical images of cell structures, enabling colocalization of α‐ and ^7^Li‐tracks with cellular compartments. This relatively simple and affordable method provides qualitative information on whether boron carriers reach radiosensitive subcellular regions, making it a useful research tool for drug development, though not readily translatable to clinical workflows [86].

HRQAR, while conceptually similar, typically involves samples mounted on Mylar (biaxially oriented polyethylene terephthalate) irradiated under vacuum conditions, with alpha spectra captured using silicon detectors. This technique allows precise localization of ^10^B within tissue sections but requires stringent experimental setups and is less suitable for high‐throughput or clinical workflows [40].

While autoradiographic methods are inherently destructive and require elaborate sample preparation, including cryosectioning, resin embedding, and precise mounting, their spatial resolution remains unmatched. Though not yet translatable to routine clinical diagnostics, these autoradiographic techniques remain indispensable in preclinical research, especially in validating the intracellular and intratumoral distribution of emerging boron carriers and supporting the design of next‐generation BNCT agents.

In contrast, fluorescent nuclear track detectors (FNTDs) offer a non‐destructive alternative that can register the same neutron‐capture reaction products in intact biological samples, eliminating the need for labor‐intensive sectioning and preparation. FNTDs represent a novel approach for measuring the microdistribution of boron in BNCT; however, their application currently remains limited to preclinical and experimental research settings. Their key advantage over traditional autoradiography and track detectors lies in their biocompatibility, which allows living cells to be cultured directly on the detector surface. By reconstructing the trajectories of the α‐ and ^7^Li‐particles emitted from the ^10^B(*n*,α)^7^Li reaction using confocal microscopy, FNTDs make it possible to estimate the original emission sites and thereby map the subcellular distribution of boron in intact biological samples. This capability could ultimately be translated into patient‐specific assessments of boron microdistribution and radiation track structure, providing information that is currently inaccessible in the clinic. Emerging FNTD‐based biosensor platforms, such as Cell‐Fit‐HD and Cell‐Fit‐HD^4D^, further extend this concept by enabling real‐time correlation between boron localization, particle track formation, and DNA damage repair dynamics. While still at an early experimental stage, these systems illustrate how FNTD technology may inform future strategies for individualized BNCT treatment planning, although clinical implementation remains a long‐term perspective [87, 88, 89].

Neutron‐based techniques remain essential for precise boron quantification in BNCT, offering high isotope specificity and both bulk and spatially resolved measurements. PGRA continues to serve as a reference standard, with advancements in detectors and compact neutron sources enhancing its clinical potential. PG‐SPECT introduces three‐dimensional imaging, while autoradiographic methods provide unmatched (sub)cellular resolution for validating boron delivery. Despite infrastructural and workflow challenges, ongoing technological improvements are steadily increasing the clinical viability of these methods.

### 5.3 AES Techniques

AES remains a cornerstone technique in BNCT research for quantifying total boron concentrations in biological matrices. Among the most utilized methods are ICP‐AES (also known as ICP‐OES) and, to a lesser extent, DCP‐AES. These techniques operate by exciting atoms within a plasma, causing them to emit light at element‐specific wavelengths that are then detected and quantified.

ICP‐AES has been widely adopted due to its robustness, moderate infrastructure needs, and compatibility with biological samples. It is commonly applied to digested tissue, blood, urine, and pharmaceutical preparations. Recent work has emphasized optimizing sample preparation protocols to improve reproducibility and throughput. This approach provides reproducible biodistribution and pharmacokinetic data, establishing it as a standard analytical tool for evaluating boron delivery agents [90, 91]. More recently, ICP‐AES protocols were optimized and validated for boron determination in animal tumor models, demonstrating a rapid and broadly applicable approach suitable for biodistribution studies of different boron compounds [92]. In subsequent BNCT studies, ICP‐AES was employed to confirm therapeutically relevant tumor boron concentrations following BPA administration in rat glioma models, supporting the evaluation of BNCT efficacy in vivo [93].

Despite its advantages, ICP‐AES is inherently destructive, requires complete digestion of samples, and cannot differentiate between the isotopes ^10^B and ^11^B. This lack of isotope selectivity limits its ability to directly assess the therapeutic fraction of boron unless isotope enrichment is known in advance. Moreover, it lacks spatial resolution and is not suitable for real‐time or in vivo measurements.

DCP‐AES, an older technique using a direct current arc plasma, offers simpler instrumentation but suffers from poorer sensitivity and stability. It has largely been phased out of BNCT‐related applications and is rarely mentioned in recent literature, suggesting that it is now largely obsolete in this context.

**Figure 6:**
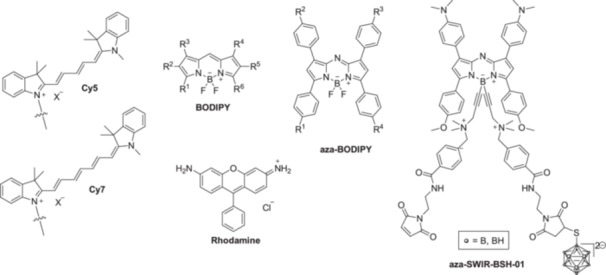
Representative fluorophores explored as optical tags in BNCT theranostics. Structures include cyanine dyes Cy5 and Cy7 (used in albumin–boron conjugates by Popova et al.), rhodamine, as well as BODIPY, aza‐BODIPY, and the near‐infrared aza‐SWIR‐SH01 dye (applied in boron–BSH conjugates by Kalot et al.) [96, 135].

LIBS has recently emerged as a rapid, minimally invasive, and versatile tool for in situ elemental profiling and imaging in biological tissues, offering ppm‐level sensitivity, microscopic resolution, and minimal sample preparation. Recent reviews highlight its ability to detect and map both endogenous and exogenous elements across plant, animal, and human specimens, underscoring its growing relevance for preclinical research and potential future medical diagnostics [94]. In LIBS, a high‐energy pulsed laser ablates a microscopic amount of sample material, producing a transient microplasma whose atomic emission lines are spectrally analyzed for elemental identification and quantification. Like ICP‐AES/OES and DCP‐AES, LIBS relies on characteristic optical emission, but it generates the plasma directly on the sample surface rather than in an inductively coupled or direct current plasma. The technique offers several advantages for BNCT workflows, including minimal sample preparation (room temperature and atmospheric pressure), multi‐element capability, and spatially resolved measurements [95]. In preclinical BNCT models, fluorescent aza‐BODIPY (shown in Figure 6) boron compounds enabled in vivo optical imaging of tumor uptake, while ex vivo elemental mapping with LIBS confirmed boron biodistribution, highlighting the complementarity of the two approaches [96]. More broadly, recent reviews have emphasized methodological advances in LIBS for biomedical and analytical applications. Jantzi et al. highlighted how tailored sample preparation strategies can markedly improve sensitivity and reproducibility, while Leprince et al. focused on LIBS‐based elemental imaging of biological tissues, underscoring its potential for preclinical and medical studies at trace concentration levels [97, 98].

Although LIBS remains primarily a laboratory‐based technique, its portability, rapid acquisition times, and compatibility with solid biological samples make it a promising adjunct for BNCT research, particularly for validating boron delivery agents and correlating with histological findings [87, 99]. However, LIBS remains largely preclinical in the BNCT field, with challenges including matrix effects, lower sensitivity compared to ICP‐based methods, and the need for calibration curves based on suitable standards, which are difficult to generate for biological materials [87].

In conclusion, while ICP‐AES remains a mainstay in boron quantification workflows due to its ease of use, analytical accuracy, and compatibility with existing laboratory infrastructure, it does not provide isotopic or spatial resolution. Recent refinements in sample preparation and digestion have further improved its reliability, particularly for pharmacokinetic evaluations and large‐scale screening of boron delivery systems. Complementing this, LIBS offers rapid, minimally destructive elemental analysis with the added benefit of spatial mapping and minimal sample preparation, making it a promising adjunct for preclinical BNCT studies. However, both ICP‐AES and LIBS lack isotopic discrimination. In addition, ICP‐AES requires time‐intensive sample processing, while LIBS remains predominantly a laboratory‐based method. These factors continue to limit their direct clinical integration.

### 5.4 MS‐Based Techniques

MS has become a cornerstone in boron analysis for BNCT due to its exceptional sensitivity, elemental specificity, and isotopic resolution. Among the various MS modalities, ICP‐MS remains the most established and widely applied technique for determining total boron content in biological samples. This method operates by digesting samples (Section 4) before introducing the solubilized boron into a high‐temperature plasma for ionization. The ions are then separated by a quadrupole or sector field analyzer based on their mass‐to‐charge ratio, enabling detection limits down to the parts‐per‐trillion range and the ability to distinguish between ^10^B and ^11^B [55, 100].

Recent efforts have improved both sample preparation and analytical throughput. For example, combined acid and UV digestion protocols have been optimized for ICP‐MS analysis of cell cultures, achieving full boron recovery while minimizing spectral interferences [55]. Alternative MS‐based approaches are gaining traction. UHPLC‐MS/MS has been established as a robust method for quantifying boronated compounds such as BPA in biological matrices, offering high specificity and sensitivity for pharmacokinetic studies and complementing traditional ICP‐based analyses [49, 101].

Although they are destructive and lack spatial resolution, these MS techniques remain indispensable in preclinical and translational BNCT research due to their widespread availability, robust quantification capabilities, and ability to discriminate between isotopes. However, their reliance on centralized laboratory infrastructure and time‐intensive sample preparation currently limits their clinical deployment. In addition, ICP‐MS analysis of boron can be affected by memory effects and carry‐over phenomena, requiring rigorous washout protocols and careful quality control to ensure accurate quantification, thereby potentially limiting throughput in routine clinical settings.

**Figure 7:**
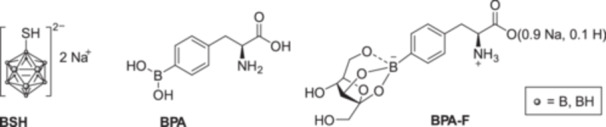
Structures of sodium mercaptoundecahydro‐closo‐dodecaborate (BSH), 4‐borono‐L‐phenylalanine (BPA), and its fructose complex (BPA‐F) [103].

To resolve the spatial heterogeneity of boron at cellular and subcellular levels, advanced MSI techniques have become essential in BNCT research. SIMS and its high‐resolution variants, nanoSIMS and ToF‐SIMS, use a FIB (e.g., Cs⁺, O⁺) to sputter atoms from cryogenically prepared samples, detecting secondary ions to map elemental distributions. SIMS offers micrometer‐scale resolution, while advanced variants such as nanoSIMS can achieve lateral resolution below 50 nm, enabling isotopic imaging of ^10^B and ^11^B within organelles and even nuclear compartments [42, 102]. These methods require precise sample preparation, including cryosectioning, freeze‐drying, and conductive coating, and operate under ultra‐high vacuum, which limits their throughput and clinical adaptability [40]. Complementing this, laser‐SNMS combines SIMS‐like sputtering with laser‐induced post‐ionization to reduce matrix effects and enhance sensitivity. Using this approach, laser‐SNMS enables detection of boron at low concentrations in single cells and allows differentiation of localization patterns of BPA and BSH (sodium mercaptoundecahydro‐*closo*‐dodecaborate, Figure 7), including nuclear uptake of both agents, with BSH distributing boron homogeneously and BPA more heterogeneously [104]. MSI techniques such as MALDI‐MSI extend chemical mapping beyond SIMS by enabling spatially resolved detection of larger biomolecules, with recent advances achieving cellular and even subcellular resolution in intact tissues [102].

LA‐ICP‐MS enables quantitative mapping of boron by ablating tissue regions and analyzing the aerosol in ICP‐MS. In BNCT studies, it has been applied to visualize ^10^B distribution within bone structures such as growth cartilage, trabecular bone, and marrow, providing spatial context to complement bulk concentration measurements [69]. Sample preparation typically involves cryosectioning or resin embedding and mounting to ensure precision and repeatability.

Although destructive and infrastructure‐intensive, these MSI modalities remain indispensable in preclinical research. They provide critical quantitative and spatial data to validate emerging boron delivery agents, support mechanistic drug development, and refine dosimetry models.

### 5.5 Nuclear Imaging Techniques

PET has become a cornerstone in BNCT for non‐invasive, real‐time evaluation of boron agent biodistribution in vivo. PET relies on the detection of pairs of 511 keV annihilation photons emitted when positrons, released from radionuclides, encounter electrons in tissue. A variety of isotopes are used in this context, most commonly ^18^F (*t*_½_ = 109.7 min), ^64^Cu (*t*_½_ = 12.7 h), ^68^Ga (*t*_½_ = 67.7 min), ^89^Zr (*t*_½_ = 78.4 h), and ^124^I (*t*_½_ = 100.2 h), each differing in half‐life, positron energy, and radiochemistry, thereby allowing adaptation to diverse biological targets and imaging time windows [105]. More recently, ^52^Mn (*t*_½_ = 5.6 days) has gained attention as a dual‐modality isotope combining PET sensitivity with intrinsic MRI contrast, highlighting its promise for future multimodal theranostics. Among these isotopes, ^18^F remains the most widely applied due to its favorable half‐life and radiochemical versatility [106, 107, 108].

**Figure 8:**
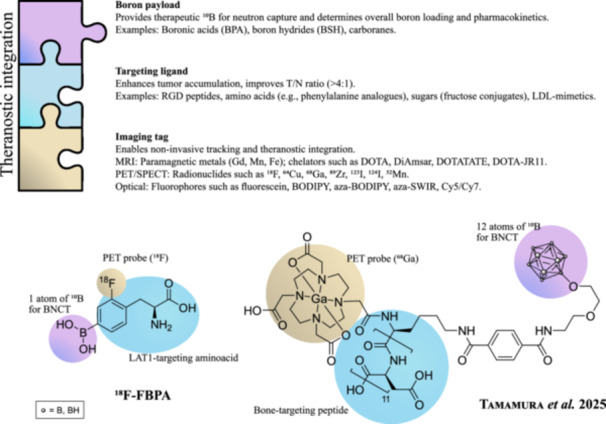
Schematic representation of the theranostic pharmacophore model in BNCT, consisting of three functional components: (1) the boron delivery moiety (purple), providing the therapeutic payload; (2) a tumor‐targeting ligand (blue), enhancing accumulation and T/N tissue selectivity; and (3) an imaging tag (yellow), enabling non‐invasive monitoring of biodistribution. The concept is illustrated with two representative examples: ^18^F‐FBPA, the current clinical PET standard, and the closo‐dodecaborate‐Ga‐DOTA conjugate by Tamamura et al., which integrates a boron payload, a bone‐targeting unit, and a gallium‐based imaging probe [96, 154]. [Color figure can be viewed at wileyonlinelibrary.com]

Within BNCT research, ^18^F‐FBPA (shown in Figure 8), a radiolabeled analog of the therapeutic boron compound BPA, has emerged as the key clinical tracer. Following intravenous administration, ^18^F‐FBPA mimics the pharmacokinetics of ^10^B‐BPA, enabling clinicians to assess T/N and T/B uptake ratios, which are critical metrics for patient selection and treatment planning. Recent clinical advances have enhanced the utility of ^18^F‐FBPA PET. LAT1 and CD98, the principal transporters for BPA and ^18^F‑FBPA, have been investigated as biomarkers for BNCT candidate selection [109]. Matović et al. showed that immunohistochemical assessment of these markers can improve predictions of therapeutic outcome, supporting their integration with molecular imaging like ^18^F‑FBPA PET in future workflows [110, 111]. Furthermore, Kashihara et al. demonstrated a moderate positive correlation between LAT1 expression and ^18^F‑FBPA uptake in head and neck cancer patients, reinforcing the biological specificity of this imaging technique [112]. Clinically, ^18^F‑FBPA PET continues to serve as a non‐invasive tool for estimating T/N and T/B uptake ratios essential for BNCT treatment planning. Recent technical advances, such as the implementation of PET/MR with zero‐echo‐time attenuation correction, have improved quantification accuracy of T/N metrics, bringing PET more reliably into treatment workflows [113]. Romanov et al. highlighted the capabilities of total‐body PET systems to reduce scan times, improve sensitivity, and permit lower tracer doses [114].

Dynamic ^18^F‐FBPA PET with compartmental modeling can be used to estimate tissue boron concentrations prior to BNCT. In standard BNCT treatment planning, dose distributions are calculated using Monte Carlo neutron transport simulations. With this in mind, some authors attempted to use the patient‐specific boron concentrations derived from PET to calculate the boron dose component. However, it should be noted that PET tracers are administered at micro‐ to milligram levels, whereas therapeutic BNCT involves gram‐scale administration of boron agents (10–20 g). This substantial discrepancy introduces a pharmacokinetic scaling challenge, as tracer‐based biodistribution may not fully reflect therapeutic conditions. Consequently, PET primarily provides relative distribution patterns rather than direct therapeutic concentrations, and absolute dose estimation requires pharmacokinetic modeling, scaling, and additional validation [23, 44, 95].

Beyond ^18^F‐FBPA, novel PET tracers such as ^18^F‐NaBF₄ are introduced as a promising candidate for thyroid‐targeted BNCT due to its sodium‐iodide symporter (NIS)‐mediated uptake and in vivo stability [115]. This tracer enables reliable estimation of boron concentrations using PET‐derived standardized uptake values, potentially supporting new clinical applications. Beyond its role as an experimental imaging agent, ^18^F‐NaBF_4_ also illustrates how PET tracers may be incorporated into theranostic BNCT strategies, a topic further discussed in Section 6.

Complementing clinical research, preclinical studies underscore the importance of tracer kinetics in matching the temporal dynamics of neutron irradiation. Optimized PET protocols for monitoring boron delivery profiles, facilitated by the proliferation of compact cyclotron systems and automated radiochemistry platforms, enhance tracer accessibility [116]. Further innovation includes multimodal imaging strategies like a PET/optical dual‐mode probe for simultaneous macroscopic and microscopic tracking of boron compounds [117].

While PET excels in sensitivity and whole‐body imaging, it remains limited to indirect quantification of boron compounds. The need for on‐site cyclotron infrastructure and the short half‐life of ^18^F (~110 min) remain logistical challenges. Nonetheless, ^18^F‐FBPA PET is widely regarded as the non‐invasive standard for evaluating boron delivery and remains integral to individualized BNCT treatment planning.

SPECT has occasionally been used in BNCT research when boron delivery agents are labeled with conventional γ‐emitters (e.g., ^99m^Tc, ^123^I, ^125^I, ^111^In), enabling preclinical biodistribution studies rather than monitoring during therapy [105, 118, 119]. However, this conventional SPECT approach is experimentally rare and adds complexity by requiring dual‐purpose probes, unlike PG‐SPECT, which directly leverages the boron isotope's neutron‐capture γ‐signal for in‐treatment imaging [57, 72, 73, 120]. For a comprehensive discussion of how PG‐SPECT fits within neutron‐based dosimetry and hardware innovation, see Section 5.2; and Section 6 details how tracer design integrates imaging and therapy in theranostic strategies.

In summary, nuclear imaging techniques in BNCT differ fundamentally in analytical principle and clinical applicability. PET provides highly sensitive, non‐invasive, whole‐body imaging with excellent temporal resolution, but it does not measure boron directly; instead, it relies on radiolabeled surrogates whose pharmacokinetics approximate those of therapeutic boron carriers. This indirectness, together with the need for isotope‐production infrastructure, limits absolute quantification, but it still makes PET the most clinically integrated method for treatment planning. Conventional SPECT, while technically feasible when boron carriers are co‐labeled with γ emitters, has little practical role beyond preclinical probe evaluation, as the addition of a secondary radionuclide undermines its utility in BNCT. In contrast, PG‐SPECT directly exploits the neutron capture reaction of ^10^B itself and is therefore conceptually distinct from tracer‐based imaging. Its technical basis and current translational limitations are discussed in Section 5.2, while its theranostic role is considered in Section 6.

### 5.6 Spectroscopic and Magnetic Techniques

Magnetic resonance‐based techniques have emerged as valuable analytical tools in BNCT, offering non‐destructive chemical analysis and, in specific contexts, in vivo imaging capabilities. In the context of this review, these approaches are considered primarily from the perspective of their ability to enable or support quantitative assessment of boron distribution in vivo, rather than from a drug design point of view. Among these, NMR spectroscopy plays a foundational role in characterizing boron speciation. Both ^10^B and ^11^B isotopes are NMR‐active; ^11^B, with a spin of ^3^/_2_, is more commonly used due to its higher sensitivity, while ^10^B, with a spin of 3, has lower sensitivity and yields broad signals, but enables direct investigation of the therapeutic isotope.

A notable application of ^10^B‐NMR in the context of BNCT has been explored as a non‐destructive method to distinguish and quantify different boron species in blood, including ^10^B‐BPA, ^10^B‐BPA–fructose complexes, and total ^10^B. Using extended signal accumulation, detection limits of 3–6 ppm were achieved without chemical degradation of the analytes. However, acquisition times on the order of 100 min per sample and the requirement for high‐field NMR infrastructure currently limit the clinical applicability of this technique [121].

MRI is increasingly investigated in BNCT, both as a clinical tool for anatomical and physiological imaging and as a platform for theranostic agent development [122]. Direct detection of boron nuclei is technically challenging: ^11^B MRI has been demonstrated experimentally but remains limited by sensitivity and relaxation properties, while ^19^F‐MRI using fluorinated BPA analogs has shown promise in preclinical models for mapping boron drug uptake. More clinically advanced strategies rely on indirect imaging through conjugation of boron carriers to paramagnetic contrast agents such as gadolinium (and to a far lesser extent manganese), enabling simultaneous visualization of boron biodistribution and enhancement of conventional T_1_‐weighted MRI contrast [108, 116]. This dual functionality enables indirect tracking of boron biodistribution in vivo, while simultaneously providing critical anatomical and physiological information relevant to BNCT planning. While gadolinium‐based contrast enables such indirect visualization, the incorporation of Gd introduces considerations regarding long‐term retention and potential nephrotoxicity, which remain under regulatory scrutiny and must be carefully evaluated in theranostic agent design. Representative examples are highlighted here to illustrate how MRI‐visible boron probes can support indirect quantification and biodistribution assessment. Their broader theranostic implications are discussed in detail in Section 6. These developments are particularly relevant for BNCT quantification, as they establish a measurable relationship between imaging signal intensity and boron concentration, thereby enabling indirect but spatially resolved assessment of boron distribution.

Several preclinical studies have demonstrated that gadolinium‐containing boron constructs can enable indirect, spatially resolved assessment of boron biodistribution by correlating MRI contrast with boron payload. Examples include LDL‐targeted boron–gadolinium probes in mesothelioma and melanoma models, RGD‐conjugated Gd‐boron silica nanoparticles (NPs) in glioblastoma, and albumin‐ or NP‐based Gd/boron systems that allowed non‐invasive visualization of tumor uptake and longitudinal monitoring of probe retention [25, 123, 124, 125, 126, 127, 128, 129]. These studies are relevant from an analytical perspective because they show how surrogate MRI signal can be linked to boron distribution in vivo, albeit indirectly and with system‐specific calibration. Complementary physiological MRI approaches, such as arterial spin labeling, have also been explored to monitor treatment‐related changes in tumor perfusion, thereby adding functional information to boron imaging workflows [130].

The value of MRI in BNCT lies in its ability to guide compound development, confirm tumor selectivity, and potentially enable non‐invasive boron quantification through relaxation rate mapping (i.e., creating quantitative maps of MRI relaxation times whose changes can be correlated with the local concentration and distribution of boron compounds) [131]. However, it does not directly detect boron or distinguish isotopes, and its quantitative accuracy depends on the correlation between Gd signal intensity and boron content.

The broader clinical implications and theranostic integration of MRI‐based approaches will be further detailed in Section 6, where the potential for combined diagnosis, treatment, and monitoring is critically examined.

NIRF imaging has emerged as a powerful optical modality for non‐invasive visualization of molecular probes in vivo. In the NIR‐I window (650–950 nm), fluorescence imaging achieves better tissue penetration than visible light, while the NIR‐II window (1000–1700 nm), as well as the short‐wave infrared (SWIR; 1000–2500 nm), offer further reductions in scattering and autofluorescence, enabling deeper, higher‐resolution imaging. Recent reviews of small‐molecule fluorophores highlight the diverse scaffolds available for NIR imaging, from BODIPY and aza‐BODIPY derivatives to cyanine and rhodamine‐based dyes, many of which are adaptable for biomedical applications [132, 133, 134]. In the context of this review, these probes are considered primarily for their ability to support optical tracking of boron carrier biodistribution rather than as a comprehensive class of delivery systems. Within BNCT research, cyanine, BODIPY, and aza‐BODIPY fluorophores have been incorporated into boron‐containing constructs to enable simultaneous optical tracking and therapeutic delivery. Representative examples include gold NP conjugates, carborane‐containing polymeric micelles, and phenylboronic acid–functionalized BODIPY probes, all of which demonstrated the feasibility of monitoring tumor uptake and biodistribution in preclinical models [25, 96, 135, 136, 137]. From the perspective of boron quantification, the main value of these systems lies in their ability to provide sensitive, real‐time optical readouts of probe localization, although only at limited tissue depths.

Collectively, these studies illustrate the potential of NIRF probes to integrate boron delivery with real‐time, non‐invasive imaging. While diverse scaffolds such as BODIPY, aza‐BODIPY, and cyanine dyes have demonstrated promising results in preclinical BNCT models, translation into routine clinical workflows remains limited by depth penetration, probe pharmacokinetics, and regulatory hurdles. Nonetheless, their development underscores the growing role of optical imaging in theranostic BNCT strategies and provides a foundation for future clinical translation.

In addition to NMR, MRI, and NIRF, spectroscopic techniques such as ESR/EPR and FT‐IR have been explored in BNCT research for investigating free radical formation and boron compound characterization, respectively. While these methods offer chemical specificity and structural insight, their application remains largely confined to pre‐clinical and mechanistic studies due to limited sensitivity and clinical relevance.

In summary, magnetic resonance‐based and spectroscopic techniques provide valuable insights into the chemical, structural, and spatial properties of boron agents in BNCT. NMR spectroscopy offers non‐destructive, isotope‐specific analysis, but remains constrained by low sensitivity, long acquisition times, and relatively high sample requirements, limiting its role to preclinical studies. MRI, by contrast, is clinically mature and widely available, and, when boron carriers are conjugated to paramagnetic agents, it enables indirect visualization of biodistribution and longitudinal monitoring, though without isotope specificity. NIRF imaging adds high‐sensitivity, real‐time tracking in small animal models using low‐cost equipment, but suffers from shallow penetration and lacks clinical translation to date. ESR and FT‐IR remain confined to mechanistic studies, offering insight into radical processes and molecular structure, but with little in vivo applicability. Collectively, these techniques contribute uniquely to BNCT research by enabling chemical characterization, indirect or direct analysis of boron distribution, and functional monitoring across different spatial scales. However, significant barriers remain in translating most of these tools into routine clinical use, particularly with respect to isotopic resolution, real‐time imaging, and non‐invasive, high‐sensitivity detection in deep tissues. Continued development of multimodal, theranostic platforms is therefore of particular interest not only for drug delivery but also for improving the accuracy, spatial resolution, and non‐invasive quantification of boron in clinical BNCT workflows.

### 5.7 Advanced Microscopy and Ion Beam Techniques

A variety of advanced microscopy and ion beam‐based techniques have been applied in BNCT research to study boron distribution in tissues and cells. While many of these methods offer high spatial resolution or chemical specificity, none are currently suitable for clinical routine application.

TEM combined with EELS enables high‐resolution elemental mapping based on core‐electron excitations. In the context of BNCT, this approach has been used to identify subcellular boron distribution in tissues treated with boron carriers [138]. Detection limits of EELS for boron in biological samples were reported around 0.2% (2000 ppm) with 10% accuracy, with spatial mapping feasible down to 0.5% (5000 ppm) at a pixel size of 66 nm, though limited by electron beam damage and strong carbon matrix background [139]. These methodological aspects and their critical evaluation have been extensively reviewed in the literature [40].

Beyond BNCT, developments in the broader EELS field highlight future potential. For example, Leapman et al. demonstrated three‐dimensional tomographic imaging of light elements in biological specimens using energy‐filtered TEM, while later reviews of microscopy and spectroscopy methods reported that modern EELS approaches are approaching near single‐atom sensitivity [140, 141]. More recent methodological studies confirm that low‐voltage EELS enables light‐element detection with reduced beam damage, while refined multi‐linear least squares approaches have improved accuracy and pushed detection limits into the sub‐0.1% range [142, 143]. Hachtel et al. first demonstrated isotope sensitivity by distinguishing ^12^C/^13^C and ^1^H/^2^H in molecular samples using vibrational EELS, while subsequent work with monochromated instruments has extended the approach toward complex systems, including beam‐sensitive biomaterials, enabling spatially resolved isotopic mapping at the nanometer scale [144, 145]. These advances raise the possibility that future improvements could extend to medically relevant isotopes, including ^10^B/^11^B, which would be highly relevant for BNCT.

While no substantial BNCT‐related progress in TEM/EELS has been reported since the early 2000s, advances in instrumentation and analysis methods suggest that its sensitivity and applicability may improve in the future. At present, however, the technique remains confined to preclinical and mechanistic studies: it requires elaborate and time‐consuming preparation of ultrathin cryo‐sections, is highly invasive and beam‐sensitive, and still lacks boron isotope discrimination, all of which preclude clinical integration [40].

STIM provides topographic and density mapping by measuring the energy loss of MeV ions as they traverse biological samples. While not element‐specific, STIM is often paired with PIXE to provide structural context and correct for thickness‐dependent effects. PIXE is a well‐established method for trace elemental analysis, and when implemented in microbeam setups (micro‐PIXE), it enables mapping of boron and related compounds at micrometer resolution. Using this approach, Stoliar et al. employed a heavy‐ion microprobe to generate two‐dimensional concentration maps of a copper‐containing boronated porphyrin (CuTCPH) in tumor‐bearing hamsters, revealing highly heterogeneous compound distribution across tissue sections. As emphasized in a recent review by kreiner, such findings illustrate both the power and the limitations of micro‐PIXE: while it provides ppm‐level sensitivity and detailed microdistribution data, the patchy distribution of boron carriers requires sampling of multiple tumor regions to obtain a representative picture (sampling bias) [81, 146]. The technique's sensitivity reaches the low µg/g range, and it allows for simultaneous multi‐elemental analysis. Importantly, PIXE is non‐destructive and has been successfully combined with STIM for quantitative boron mapping. However, its main limitations are the inability to differentiate boron isotopes and the need for vacuum‐compatible sample preparation, which hinders direct in vivo or real‐time clinical use.

Whereas PIXE and related ion beam methods provide high‐resolution elemental maps, synchrotron‐based spectroscopic approaches such as XAS focus on the chemical speciation of boron, offering complementary insights into its metabolic fate and bonding environment.

XAS, encompassing X‐ray absorption near‐edge structure (XANES) and extended X‐ray absorption fine structure (EXAFS), probes the local chemical environment of atoms by analyzing how X‐ray absorption varies near and beyond an absorption edge. XANES provides information on oxidation state and bonding geometry, while EXAFS reveals the radial distribution of neighboring atoms.

In BNCT research, early applications of synchrotron‐based XANES (e.g., with the MEPHISTO spectromicroscope, Microscope à Emission de Photoélectrons par Illumination Synchrotronique de Type Onduleur = Photoelectron Emission Microscope by Synchrotron Undulator Illumination) demonstrated that boron compounds such as BSH could be detected and distinguished in glioblastoma tissues, revealing altered chemical states after in vivo metabolism. Related studies in rat brain tumors confirmed preferential boron uptake in tumor tissue, though with pronounced heterogeneity at the micrometer scale [147, 148]. More recently, XANES and EXAFS have been applied primarily to NP carriers, for example, to characterize Au‐B NPs and confirm their short‐range atomic structure, stability, and potential as dual BNCT/XRT vectors [149].

A complementary synchrotron‐based method, scanning transmission X‐ray microscopy (STXM), raster‐scans samples with monochromatic X‐rays to acquire absorption contrast together with localized XRF or XANES spectra. As highlighted by Bonanni et al., STXM offers the unique advantage of combining sub‐micrometer imaging with chemical speciation, while requiring less elaborate sample preparation than TEM–EELS and providing higher sensitivity to biologically relevant elements such as C, N, O, S, and Ca. However, its limitations, like restricted sample thickness, limited accessible energy ranges, and relatively long acquisition times, constrain current applications largely to in vitro or ex vivo studies.

Advanced microscopy and ion beam‐based techniques have provided invaluable insights into boron distribution at cellular and subcellular scales, often reaching spatial resolutions that surpass other analytical methods. TEM/EELS and related electron spectroscopies offer near‐nanometer mapping but require ultrathin cryo‐sections, are beam‐sensitive, and still lack isotope discrimination. Ion beam methods such as micro‐PIXE deliver quantitative, non‐destructive elemental maps with ppm sensitivity, but depend on vacuum preparation and cannot differentiate ^10^B from ^11^B, limiting clinical translation. Synchrotron‐based XAS and STXM add unique chemical speciation and structural information, yet their reliance on large‐scale facilities and long acquisition times confines them to preclinical or drug development contexts.

Collectively, these methods remain powerful research tools for validating new boron carriers and exploring mechanistic questions in BNCT. However, none currently meet the requirements of routine clinical workflows, where real‐time, non‐invasive, and isotope‐specific imaging in intact patients will be essential.

### 5.8 Other Quantification Techniques

CPAA and PIGE are quantitative, bulk analytical techniques employed in BNCT research for the accurate determination of ^10^B concentrations in biological samples. These methods are best suited to pharmacokinetic assessments and treatment verification, rather than imaging or spatial mapping.

In CPAA, samples are irradiated with 8 MeV protons to induce the ^10^B(p,α)^7^Be reaction, and the resulting 478 keV γ‐rays from ^7^Be decay are measured against internal standards (e.g., ^56^Co from blood iron) to quantify boron with high isotopic specificity. CPAA achieves excellent linearity (r > 0.999) across 3–300 µg/g and requires minimal preparation, preserving the chemical integrity of the sample. Its limitations are long analysis times, reliance on accelerator and γ‐ray spectroscopy infrastructure, and lack of spatial or real‐time capability [36]. PIGE, in contrast, detects prompt γ‐rays at 428 keV from the ^10^B(p,p′γ)^7^Be reaction, enabling direct mapping of boron in biological specimens. When implemented in microbeam setups (micro‐PIXE/PIGE), it has been used to visualize intracellular boron after BPA exposure, showing quantifiable differences between exposed and control cells. While promising for mechanistic studies, PIGE faces the same challenges as CPAA in terms of clinical integration: accelerator dependence, limited availability, and no true in vivo compatibility [150].

Recent work outside the biomedical field shows that CPAA methodology continues to evolve, for example, through the development of graphite reference materials enabling boron quantification at detection limits of 1.4 mg/kg. While this illustrates the robustness of CPAA as an ultra‐trace analytical tool, such advances have not translated into clinical BNCT, where biological sample analysis remains the primary concern [151]. For PIGE, technical progress has been more directly relevant. A standardized “external” PIGE approach has been proposed that uses atmospheric nitrogen as an internal normalizer, allowing nondestructive, cost‐effective boron quantification without dedicated beam monitoring equipment. Although such methodological refinements strengthen the analytical reliability of PIGE, the technique still faces fundamental barriers to clinical adoption, notably its dependence on accelerator facilities and incompatibility with real‐time, in vivo workflows [152].

In summary, CPAA and PIGE provide highly accurate bulk quantification of boron and are particularly well suited to pharmacokinetic studies and treatment verification. CPAA offers excellent isotopic specificity and precision but is constrained by long acquisition times, reliance on accelerator infrastructure, and the absence of spatial or real‐time information. PIGE, in contrast, enables faster measurements with minimal sample preparation and limited mapping capability, but lacks isotope discrimination and remains dependent on microbeam facilities. Both techniques are essentially non‐destructive, yet their bulk nature precludes cellular resolution, and their infrastructure requirements prevent routine use in clinical workflows. Recent refinements, such as high‐linearity CPAA for whole‐blood and “external” PIGE using atmospheric nitrogen as an internal normalizer, underscore ongoing technical progress, although translation into clinical practice remains unlikely [36, 150, 152].

## 6 Theranostics and Multimodal Imaging

Theranostics, the integration of diagnostic imaging and targeted therapy, has become a cornerstone in the evolution of BNCT.

By combining patient‐specific imaging with therapeutic delivery in a single workflow, theranostics offers a pathway to individualized treatment planning, precise dose verification, and real‐time monitoring of therapeutic efficacy. In this review, theranostic approaches are considered specifically in terms of their contribution to the quantification, spatial mapping, and clinical measurement of boron distribution, rather than as a comprehensive overview of boron delivery‐agent design. Whereas Section 5 describes the underlying analytical and imaging methods, the present section focuses specifically on how these modalities are integrated with boron delivery systems to enable combined measurement, targeting, and therapeutic monitoring. The 2021 review by Sauerwein et al. summarized the state of the art, establishing ^18^F‐FBPA PET as the gold standard for clinical boron imaging, highlighting early developments in MRI‐visible boron carriers, reporting on optical probes in preclinical studies, and introducing the first generation of multimodal NP platforms [95]. Since then, rapid progress has been made in probe chemistry, multimodal integration, and imaging instrumentation, with an increasing emphasis on technologies that can be translated into clinical BNCT practice [25]. Building on the clinical role of ^18^F‐FBPA PET outlined in Section 5.5, recent theranostic developments have focused on extending PET from biodistribution assessment toward probe‐integrated quantification and multimodal treatment guidance [25, 105, 109, 153]. From a quantification perspective, these developments are particularly important because they define how accurately imaging‐derived signals can be translated into estimates of boron concentration in vivo. Building on this foundation, recent developments have introduced new tracers and optimized protocols that extend the diagnostic reach of PET and begin to align imaging with therapeutic targeting [105, 153].

This highlights how next‐generation tracers can bridge diagnostic imaging and therapy within a unified theranostic framework [49, 116]. Among recent developments, Tamamura et al. introduced a *closo*‐dodecaborate‐Ga‐DOTA conjugate as a theranostic prototype featuring an aspartic acid peptide for bone targeting (Figure 8). In their proof‐of‐concept work, the complex was radiolabeled with ^67^Ga, enabling preclinical biodistribution studies via SPECT. However, the ultimate goal is translation to ^68^Ga, whose short half‐life and PET compatibility would provide clinically relevant, real‐time monitoring of boron biodistribution while simultaneously delivering the therapeutic payload [154]. Whole‐body PET has recently emerged as an important technological advance in molecular imaging, offering higher sensitivity, reduced scan times, and the ability to lower tracer doses while still achieving whole‐body dynamic acquisitions. These capabilities are particularly attractive for BNCT theranostics, where accurate, time‐resolved monitoring of boron carriers is critical. However, clinical translation is limited by the very high cost of commercial whole‐body PET systems, largely driven by the expense of lutetium‐based scintillators such as LYSO (lutetium yttrium oxyorthosilicate), which account for a major fraction of total system cost [153]. To address these barriers, the Jagiellonian PET (J‐PET) consortium has pioneered the use of long, modular plastic scintillator strips. Although plastic reduces detection efficiency compared to crystals, the strips provide excellent ToF resolution, with coincidence resolving times of about 0.27 ns, because of their rapid scintillation response and fast timing. This level of timing precision supports quantitative PET imaging while keeping system costs and complexity manageable [155, 156, 157]. Recent developments demonstrated the feasibility of J‐PET for dynamic imaging and kinetic modeling of short‐lived isotopes, with simulations and prototype studies showing that its sensitivity could approach that of crystal‐based scanners [158, 159, 160]. The lower price of J‐PET makes it a potential solution for wider adoption of PET in BNCT centers, where the dual need for specialized neutron facilities and advanced imaging infrastructure has so far restricted accessibility.

While PET remains the most clinically established modality for indirect boron imaging, its dependence on short‐lived radiotracers and cyclotron access limits broader applicability. MRI, by contrast, offers non‐radioactive imaging with excellent anatomical detail and becomes theranostically relevant when boron carriers are coupled to paramagnetic probes, providing a promising complement to PET. Such multimodal constructs are especially relevant in the context of boron quantification, as they aim to establish predictable relationships between imaging contrast and boron payload.

MRI‐guided BNCT has gained traction through the development of gadolinium‐containing theranostics, which combine boron delivery with established MRI contrast capabilities. Okada et al. introduced a gadolinium–boron albumin conjugate (Gd‐MID‐BSA), which achieved high tumor uptake in murine models and enabled non‐invasive visualization of biodistribution via T_1_‐weighted MRI. Importantly, boron and gadolinium concentrations exceeded therapeutic thresholds, and subsequent neutron irradiation produced significant tumor suppression without apparent side effects, underscoring its translational potential for MRI‐guided treatment planning [129]. Similarly, Wu et al. developed gadolinium‐ and boron‐loaded polymer–gold NPs (PLGA‐Gd/B‐AuNPs), which provided robust MRI contrast and correlated Gd and boron uptake, allowing simultaneous tracking of probe distribution and therapeutic payload. In vivo, these NPs demonstrated clear tumor visualization and favorable T/N ratios, highlighting their promise as bimodal imaging and therapy agents [127].

Building on gadolinium‐based strategies, iron‐containing NPs represent another promising class of theranostic agents. Torresan et al. introduced Fe‐B NPs that combine a high boron payload with intrinsic MRI visibility, thereby enabling simultaneous tracking and delivery within a single platform [161]. In addition, iron‐based carriers offer the option of magnetic guidance and can be used to enhance boron accumulation at tumor sites through external magnetic fields [162, 163]. Beyond their role in BNCT imaging and targeting, magnetic NPs have also been explored in other therapeutic contexts, such as hyperthermia or magnetically assisted chemotherapy [164, 165, 166].

**Figure 9:**
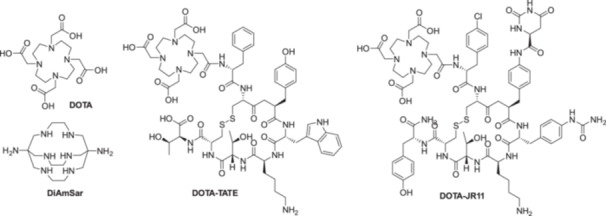
Molecular structures of representative chelators and targeting ligands with theranostic potential in BNCT. DOTA and DiAmSar are versatile macrocyclic chelators widely used for radiometals such as ^52^Mn, ^64^Cu, or ^68^Ga, enabling PET or PET/MRI dual‐modality imaging. DOTA‐TATE and DOTA‐JR11 are somatostatin analogs that combine DOTA chelation with high tumor affinity, exemplifying targeted delivery platforms that can be adapted for future boron theranostics.

Manganese‐based probes represent another conceptual extension of multimodal theranostics. In particular, the positron emitter ^52^Mn (t_½_ = 5.6 days) has attracted interest for PET/MRI dual‐modality imaging, since Mn²⁺ provides intrinsic MRI contrast in addition to its suitability as a PET isotope. Recent work has demonstrated reliable cyclotron production routes and successful chelation in ligands such as DOTA, DiAmSar, and others, as well as stable incorporation into targeting peptides like DOTA‐TATE and DOTA‐JR11 (Figure 9) [106, 108]. Although no manganese‐containing BNCT probes exist so far, the long half‐life, dual imaging capability, and compatibility with established chelation strategies suggest that ^52^Mn could become a promising candidate for future theranostic BNCT platforms. However, its clinical translation will critically depend on addressing manganese's known toxicity and ensuring chelation stability over biologically relevant timescales [108].

Fluorescence imaging has also been integrated into theranostic BNCT platforms, with several groups demonstrating dual boron delivery and optical tracking in preclinical models. As outlined in Section 5.6, aza‐BODIPY–boron conjugates, Cy5/Cy7‐labeled albumin carriers, and polymeric systems incorporating cyanine or BODIPY derivatives illustrate how conventional boron agents can be transformed into multifunctional probes (shown in Figure 6).

Within a theranostic framework, such constructs enable real‐time visualization of biodistribution alongside therapeutic action, providing a sensitive complement to radiological and magnetic resonance‐based methods. However, the limited penetration depth of optical signals currently restricts clinical translation to superficial or intraoperative applications, keeping fluorescence theranostics largely within the preclinical domain.

In addition to tracer‐based theranostics, PG‐SPECT offers a fundamentally different theranostic pathway for BNCT. Rather than relying on dual‐labeled boron carriers, PG‐SPECT exploits the 478 keV photons emitted directly from the ^10^B(*n*,α)^7^Li* reaction, thereby coupling treatment and imaging in real time. This approach enables three‐dimensional monitoring of boron distributions during irradiation without modifying the therapeutic agents themselves. While still under development, PG‐SPECT exemplifies a purely treatment‐integrated theranostic modality, complementing PET, MRI, and fluorescence‐based probes by providing intra‐treatment feedback that could guide adaptive BNCT workflows.

Theranostic approaches represent one of the most promising avenues for advancing BNCT toward individualized and clinically reliable cancer therapy. PET remains the most established platform, with ^18^F‐FBPA already embedded in clinical workflows and newer tracer concepts broadening the scope of BNCT imaging toward additional biological targets and potential applications. MRI‐based theranostics, particularly Gd‐ and Fe‐conjugated boron carriers, provide complementary anatomical and functional information and enable MR‐guided treatment planning, though their translation depends on the robustness of surrogate signal‐boron correlations. Optical probes, such as NIRF dyes, demonstrate high sensitivity in preclinical models and support pharmacokinetic optimization, but their limited penetration depth confines them to early‐stage validation. PG‐SPECT, in contrast, is unique in providing real‐time imaging of the therapeutic reaction itself, directly integrating dosimetry and treatment monitoring without altering the boron carriers. Collectively, these modalities highlight a growing toolkit in which diagnostic and therapeutic functions converge. From the perspective of boron quantification, their central value lies in enabling non‐invasive, spatially resolved, and potentially real‐time estimation of boron distribution, which remains a critical unmet need in clinical BNCT. While many approaches are still preclinical, they define the framework for future measurement strategies in which imaging signals are directly linked to therapeutic boron concentrations.

## 7 Clinical Translation, Integration, and Future Perspectives

The analytical landscape for boron quantification in BNCT spans a continuum from techniques already integrated into routine clinical workflows to advanced laboratory methods that remain firmly in the research domain. Clinical viability in BNCT analytics depends not only on accuracy but also on isotope specificity (ideally ^10^B), real‐time or near‐real‐time readouts, and seamless fit with radiotherapy workflows and hospital infrastructure. To ensure consistent classification across methods, clinical readiness categories were defined based on the level of integration into routine clinical workflows, availability of supporting clinical evidence, and infrastructural requirements. “Established” methods are those that are routinely applied in clinical BNCT workflows or closely related clinical settings, with standardized protocols and demonstrated clinical utility. “Transitional” methods are technically validated and, in some cases, applied in specialized centers, but are not yet widely implemented due to limitations such as infrastructure requirements, indirect quantification, or incomplete standardization. “Emerging” methods remain in the experimental or prototype stage, with promising initial results but without established clinical workflows or sufficient clinical validation (Table 2) [95].

**Table 2:** Comparative clinical‐readiness matrix of analytical methods for boron quantification in BNCT, integrating sample type, workflow burden, temporal suitability, evidence base, analytical performance, and present translational status.

Method	Sample type	Workflow burden	Invasiveness	Turnaround time	Evidence/Clinical implementation	^10^B/^11^B Differentiation	Sensitivity/Resolution	Clinical notes	Clinical readiness
PET (^18^F‐FBPA, etc.)	In vivo whole‐body/tumor‐region imaging	Moderate (tracer synthesis)	Non‐invasive	Near‐real‐time; dynamic PET possible	Clinical	No (only surrogate tracer)	High sensitivity, whole‐body, mm spatial resolution	Widely implemented in Japan/Finland; key for patient selection and planning	Established
ICP‐AES/OES	Blood, urine, tissue, digested biological samples; pharmaceutical preparations	Moderate (digestion and acidification)	Invasive (requires blood/tissue)	Offline; hours	Clinical/preclinical	No	ppm–ppb sensitivity, bulk only	Historically important; robust but isotope‐blind	Transitional (pharmacokinetics, reference use)
ICP‐MS	Blood, plasma, urine, tissue; digested biological samples	Moderate–high (digestion or fluid preparation)	Invasive	Offline; hours	Clinical/translational	Yes	ppt–ppb sensitivity, bulk only	Gold standard for isotope ratio and validation; widely available	Established (central lab workflows)
UHPLC‐MS/MS, FI/ESI‐MS	Serum, plasma, urine; analyte‐specific biological fluids	Low–moderate (dilution/derivatization)	Minimally invasive (blood, plasma)	Near‐real‐time to offline; minutes–hours	Clinical/translational	Indirect (analyte‐specific, not isotopes)	nM range, analyte‐specific	Excellent for pharmacokinetics and BPA monitoring	Transitional
MRI (Gd‐boron, etc.)	In vivo anatomical/tumor‐region imaging	Moderate (probe injection)	Non‐invasive	Near‐real‐time; dynamic imaging possible	Preclinical/translational	No (indirect via surrogate signal)	mm‐scale anatomical resolution	Dual theranostic role; several preclinical successes	Transitional
SPECT (^123^I, ^99m^Tc)	In vivo biodistribution imaging; mainly preclinical models	Moderate (tracer labeling)	Non‐invasive	Near‐real‐time; dynamic imaging possible	Preclinical	No	cm–mm resolution, radionuclide‐limited	Used in preclinical probe validation; rare clinical role	Laboratory/early preclinical
PGRA/PGRS/PGAA/PGNAA	Blood, tissue, bone; bulk samples	Low–moderate (minimal handling/positioning)	Semi‐invasive (requires neutron beam)	Near‐real‐time at best; minutes–hours	Preclinical/translational	Yes	ppm sensitivity, bulk	Reference standard for boron quantification in beam facilities	Transitional (beamline dependent)
PG‐SPECT	In vivo tumor‐region imaging; also phantom, cell, and pilot biological samples	Low sample preparation; high infrastructural burden	Non‐invasive	Potentially real‐time during irradiation; currently experimental	Phantom/simulation/pilot	Yes	cm‐scale, improving	Only method that images therapeutic reaction in situ; prototypes advancing	Emerging (high translational potential)
LA‐ICP‐MS	Tissue sections, bone, ex vivo biological samples	High (cryosectioning/resin embedding)	Invasive, destructive	Offline; hours–days	Preclinical/ex vivo	Yes	µm spatial resolution, ppb sensitivity	Valuable for ex vivo validation; infrastructure‐heavy	Laboratory only
LIBS	Solid tissues, surfaces, ex vivo biological samples	Low (minimal surface preparation)	Semi‐invasive (ablation)	Near‐real‐time; minutes	Preclinical/laboratory‐based	No	ppm sensitivity, µm resolution	Portable, fast, preclinical validation of boron carriers	Laboratory/preclinical

### 7.1 Clinical Workflow Considerations and Quality Assurance in BNCT Boron Analysis

In addition to analytical performance characteristics, the successful clinical implementation of boron quantification methods in BNCT critically depends on their integration into routine hospital workflows and adherence to robust quality assurance and quality control (QA/QC) standards. BNCT workflows commonly rely on blood boron measurements obtained before irradiation and, in some protocols, during treatment to support dosimetry and pharmacokinetic adjustment [23, 44, 114, 131]. This places significant demands on sample handling, analytical turnaround time, and reproducibility (Table 2).

Pre‐analytical factors represent a major source of variability. Boron is ubiquitous in the environment, and contamination from collection materials, reagents, and laboratory ware must be carefully controlled. In addition, the timing of sample collection relative to boron agent administration is critical, as even small deviations can lead to significant differences in measured concentrations due to dynamic pharmacokinetics [23, 44].

Analytically, techniques such as ICP‐MS and ICP‐AES are subject to matrix effects, instrumental drift, and memory effects, particularly for boron, which can adsorb to sample introduction systems and lead to carry‐over between measurements [55, 100]. Rigorous washout procedures, the use of internal standards, and appropriate calibration strategies (e.g., certified reference standards, internal standards, and validated analytical procedures) are required to ensure accuracy and reproducibility. These factors can impact throughput and must be balanced against the need for timely clinical decision‐making.

Turnaround time is a key constraint in BNCT workflows. While MS‐based techniques offer high sensitivity and isotopic resolution, they typically require centralized laboratory infrastructure and sample preparation steps that may delay results. In contrast, imaging‐based methods such as PET provide near real‐time information but rely on indirect quantification and tracer pharmacokinetics [23, 95, 114]. This trade‐off highlights the need for coordinated analytical strategies that combine rapid, non‐invasive assessment with high‐precision laboratory validation.

From a QA/QC perspective, methodological consistency remains important for comparability. Variability in PET data analysis and differences in boron measurement procedures may affect the reliability of comparisons across studies and centers. Greater methodological harmonization would therefore improve confidence in clinical boron assessment [23, 131].

Overall, the integration of boron quantification into clinical BNCT requires not only accurate and sensitive analytical methods but also robust workflow design, contamination control, and standardized QA/QC procedures. Addressing these practical considerations is essential to ensure reliable, reproducible, and clinically actionable boron measurements in routine practice.

### 7.2 Clinical Implementation, Challenges, and Future Perspectives

In contrast to Sections 5 and 6, which focus on methodological principles and theranostic integration, this section evaluates the practical implementation of these approaches in clinical workflows and their present level of translational maturity.

Clinical‐ready techniques include ^18^F‑FBPA PET for non‑invasive pre‑treatment evaluation of boron biodistribution [114, 131]. However, it is important to note that PET provides indirect information based on tracer‐level pharmacokinetics, which may not fully reflect the distribution achieved during therapeutic administration of boron agents. Consequently, PET‐derived data are primarily used for relative assessment (e.g., T/N ratios) in treatment planning rather than for direct quantitative dosimetry. ICP‑MS, in turn, is used for centralized, high‑precision post‑sampling quantification (with ^10^B/^11^B discrimination) of blood and tissue boron levels [167]. Techniques such as ICP‐AES and clinical MRI are widely available in clinical settings and support BNCT workflows, but are considered transitional in this context, as they do not provide isotope‐specific or direct boron quantification without additional assumptions or probe design. MRI becomes theranostically relevant only when boron carriers are coupled to paramagnetic probes. These conjugates are currently translational, not routine [127, 130]. PET and MRI are non‑invasive, provide 3D spatial information, and can be integrated directly into patient management protocols. ICP‑MS, while destructive and invasive, offers unmatched isotopic discrimination and sensitivity for validating patient‑specific pharmacokinetics.

Transitional techniques with strong clinical potential include PG‐SPECT for in‐treatment ^10^B dose mapping (active work on detector materials, background suppression, and clinical beamline integration) [57, 81], LA‐ICP‐MS for high‐resolution ex vivo spatial mapping to validate delivery and dosimetry models [168], and multimodal theranostic nanoplatforms integrating PET, MRI, and optical imaging [49, 116]. These platforms unify pre‐treatment planning with response monitoring within a single framework. Prototype Compton and CZT/GAGG systems underscore the feasibility for clinical PG‐SPECT, whereas theranostic agents remain limited by regulatory complexity and manufacturing scalability.

Laboratory‐only methods (e.g., nanoSIMS, TEM/EELS, FT‐IR, micro‐PIXE, and autoradiography/HRQAR, with FNTDs as a promising non‐destructive variant) offer unmatched resolution or mechanistic insight but are incompatible with clinical timelines, infrastructure, or in vivo use.

Advances in boron quantification methods could be synergistically integrated with modern radiotherapy approaches like image‐guided intensity‐modulated radiation therapy (IG‐IMRT) and volumetric modulated arc therapy (VMAT). These techniques use imaging to dynamically shape and steer radiation beams with millimeter precision to match tumor contours while minimizing exposure to healthy tissue [169]. In a BNCT context, boron biodistribution maps derived from PET (e.g., ^18^F‐FBPA), which provide relative distribution patterns rather than absolute therapeutic concentrations, or from MRI with boron‐sensitive contrast, could be fused with CT‐based treatment planning tools. This would guide neutron beam targeting to regions with the highest boron concentration, essentially performing dose painting, a technique already applied in IMRT to deliver differential doses within a tumor based on biological heterogeneity [170].

However, this comparison remains conceptual and should be interpreted with caution. In contrast to photon‐based IMRT/VMAT, where dose delivery can be actively modulated by beam shaping and intensity control, BNCT dose distribution is fundamentally governed by the biological distribution of boron and the characteristics of the neutron field. As a result, current BNCT workflows do not allow direct spatial modulation of dose in the same manner, and the integration of imaging‐derived boron maps is primarily used for patient selection, treatment planning, and dosimetric modeling rather than real‐time dose sculpting. Consequently, while the concept of BNCT “dose painting” highlights a potential future direction, it remains an area of ongoing research rather than established clinical practice.

Looking ahead, PG‐SPECT represents a next‐generation opportunity, offering the potential for in‐room, real‐time verification of boron dose deposition during BNCT. Once detector systems and acquisition workflows mature, PG‐SPECT could enable adaptive therapy: confirming boron uptake before or even during irradiation and guiding live beam delivery adjustments [78].

Several challenges must be addressed to move BNCT analytical science into mainstream oncology. The first is standardization: protocols for sample preparation, imaging acquisition, and boron quantification vary widely across research centers, complicating multi‑institutional data comparison. Standardized reference materials and harmonized cross‐calibration between PET, PG‐SPECT, and MS would markedly improve multi‐center comparability [95]. The second is infrastructure: neutron sources, cyclotrons for PET tracers, and advanced detector systems are not universally available. The third is regulatory: theranostic agents must meet stringent safety and efficacy requirements before clinical deployment. Finally, speed and automation are essential. Techniques requiring lengthy sample preparation or multi‑day analysis are ill‑suited to the fast turnaround demanded in radiation oncology. Beyond technical considerations, several real‐world barriers continue to influence the clinical adoption of BNCT. The regulatory classification of accelerator‐based neutron sources remains heterogeneous across jurisdictions, with some regions still applying frameworks originally developed for nuclear reactors, leading to cautious approval pathways. In addition, reimbursement models for BNCT are not yet well established in many healthcare systems, creating economic uncertainty for clinical implementation. Finally, the historical legacy of early reactor‐based BNCT trials has contributed to persistent skepticism within parts of the oncology community. These factors, together with infrastructure requirements, help explain why clinical adoption has progressed most rapidly in countries such as Japan and Finland, where dedicated programs and regulatory pathways have been established.

In the future, the most promising developments are those that push towards non‑invasive, rapid, and preferably isotopically specific diagnostics: maturation of PG‐SPECT for in‐treatment mapping, wider access to PET via total‐body and cost‐reduced plastic‐scintillator (J‐PET) systems, and standardized multimodal theranostic platforms validated in multi‐center trials [105, 127]. Continued deployment of accelerator‐based neutron sources will be pivotal for clinical scale‐up. Exploratory tools such as FNTD biosensors provide valuable mechanistic insight into boron microdistribution and radiation track structure but remain confined to preclinical research and are not yet suitable for clinical implementation [81].

From a translational perspective, the current landscape of BNCT boron analytics can be structured into a pragmatic development roadmap. Techniques such as ^18^F‐FBPA PET and ICP‐MS are already integrated into clinical or near‐clinical workflows and represent the present standard for treatment planning and pharmacokinetic validation, despite inherent limitations such as indirect quantification in PET. A second group of methods, including PG‐SPECT, LA‐ICP‐MS, and multimodal theranostic platforms, has demonstrated technical feasibility and strong potential but requires further standardization, inter‐institutional validation, and workflow integration before routine clinical adoption can be achieved. Finally, a range of high‐resolution and mechanistic techniques, such as nanoSIMS, autoradiography, and advanced ion beam methods, remain essential for preclinical research and method development but are unlikely to translate into clinical practice in their current form.

Progress across these techniques will depend on coordinated efforts in standardization, cross‐validation, and infrastructure development, with particular emphasis on linking imaging‐derived signals to quantitatively reliable boron concentrations. In this context, the most immediate impact is expected from approaches that combine non‐invasive imaging with validated quantitative frameworks, while longer‐term advances will rely on the successful clinical translation of real‐time, isotope‐specific measurement technologies.

## Data Availability

Data sharing is not applicable to this article as no new data were created or analyzed in this study.
